# Description of two new species of Paraonidae (Annelida) from the Gulf of Thailand, Western Pacific

**DOI:** 10.3897/zookeys.951.51686

**Published:** 2020-07-22

**Authors:** Jintana Plathong, Pablo Hernández-Alcántara, Leslie Harris, Sakanan Plathong

**Affiliations:** 1 Marine Ecosearch Management Co., Ltd., 4/31 Moo 1, Namnoi, Hatyai, Songkhla, Thailand Marine Ecosearch Management Co., Ltd. Hatyai Thailand; 2 Marine Science Learning Center, Department of Biology, Faculty of Science, Prince of Songkla University, Hatyai, Songkhla, Thailand Prince of Songkla University Hatyai Thailand; 3 Unidad Académica de Ecología y Biodiversidad Acuática, Instituto de Ciencias del Mar y Limnología, Universidad Nacional Autónoma de México. Circuito Exterior S/N, Cd. Universitaria, Cd. México, México Universidad Nacional Autónoma de México Mexico City Mexico; 4 Natural History Museum of Los Angeles County, 900 Exposition Blvd. Los Angeles CA, USA Natural History Museum of Los Angeles County Los Angeles United States of America

**Keywords:** *
Aricidea
*, paraonids, polychaetes, Songkhla Sea, taxonomy

## Abstract

Two new species of *Aricidea* Webster, 1879 (Paraonidae), Aricidea (Acmira) anusakdii**sp. nov.** and Aricidea (Aricidea) thammapinanae**sp. nov.** were collected from 10–26.5 m depth, in soft bottoms with mud mixed with sand and shells at Songkhla Sea, the Gulf of Thailand between 2011–2018. Aricidea (Acmira) anusakdii**sp. nov.** is clearly distinguished from other species of the subgenus Acmira by having a rounded bilobed prostomium divided by a slight notch on the anterior margin; red pigments on the subdistal to the tip of each branchia (new character); two prebranchial chaetigers; 48–68 pairs of branchiae; and modified neurochaetae as strong curved spines with blunt shafts surrounded by pubescence from chaetigers 19–44. On the other hand, Aricidea (Aricidea) thammapinanae**sp. nov.** can be separated from other members of the subgenus Aricidea by the presence of a biarticulated median antenna; distinctive notopodial lobes as broad triangular with short distal protuberances on chaetiger 3, 4–8 pairs of branchiae; and modified neurochaetae as bidentate neurochaetae with a long pubescent subterminal arista on the concave side. All data have been archived and are freely available from the Dryad Digital Repository (https://doi.org/10.5061/dryad.hqbzkh1cn).

## Introduction

Polychaetes in the seas around Thailand are poorly known, especially those belonging to Paraonidae, a family of small burrowing polychaetes usually found in soft sediments (Rouse and Pleijel 2001). Until now, the only study of Thai paraonid species was published by Lovell (2002), who reported 19 taxa of paraonids from the Andaman Sea around Phuket Island, of which three species were newly described. The present study is the result of a monitoring program carried out between 2011 and 2018 entitled “Status of Coastal and Marine Resources and Ecosystem in Songkhla’s Sea and Monitoring projects of Petroleum Production Area in Songkhla Sea”. The family Paraonidae was one of the most species-rich families in the study area with over 20 undescribed taxa. The genus *Aricidea* is the most diverse in the Paraonidae, with more than 75 known species (Blake 2019). Strelzov (1973) divided the genus into four subgenera: *Aricidea* sensu stricto, *Aedicira* Hartman, 1957, *Allia* Strelzov, 1973 (= *Strelzovia* Aguirrezabalaga, 2012), and *Acesta* Strelzov, 1973 (= *Acmira* Hartley, 1981), which were separated based on the nature of the modified neurochaetae. Hartley (1981) pointed out that the chaetal differences are unclear to justify the generic status for the four subgenera (Blake 2019), and these subgenera have largely been accepted by taxonomists (Blake 1996, 2019; Lovell 2002; Arriaga-Hernández et al. 2013, among others). During the identification process, we observed several specimens from the genus *Aricidea* Webster, 1879 that had a combination of taxonomic characters not found in the previously described species.

At present, 19 species of the subgenus Aricidea (Acmira) have been described (Read and Fauchald 2020), but Aricidea (Acmira) simonae Laubier & Ramos, 1974 is the only species that has two prebranchial chaetigers. From the Andaman Sea around Phuket, Lovell (2002) reported five species of Aricidea (Acmira), all with three prebranchial chaetigers: Aricidea (Acmira) assimilis Tebble, 1959, A. (Acmira) catherinae Laubier, 1967 and A. (Acmira) simplex Day, 1963 and two taxa that have not yet been formally named.

Another subgenus, Aricidea (Aricidea), is characterized by the presence of cirriform prostomial antennae, usually articulated, and modified neurochaetae either pseudocompound or hooked with subterminal spines on the concave side (Blake 1996). Fifteen species have been described from various localities around the world (Table 1), but in the seas around Thailand only Aricidea (Aricidea) fragilis Webster, 1879, A. (Aricidea) multiantennata Lovell, 2002 and A. (Aricidea) thailandica Lovell, 2002 have been reported. The specimens examined in the present study were characterized by the shape of the median antenna, the structure of the modified chaetae, and particularly by the shape of the third notopodial lobe. The latter was broadly triangular with a short round distal protuberance; this feature suggested that these paraonids could belong to an undescribed species.

**Table 1.** Comparative morphological characteristics of species belonging to the subgenus Aricidea (Aricidea) Webster, 1879.

Species	Prostomium	Eyes	Antenna (end at chaetiger)	Branchiae from chaetiger	Notopodial postchaetal lobes (at body region)	Modified neurochaetae	Modified chaetae from chaetiger	Type locality
Aricidea (Aricidea) capensis Day, 1961	Elongate cone, tapered anteriorly	Absent	Long, faintly annulated; to chaetiger 2	4 to 17	Prebranchial: small. Branchial: enlarged. Posterior: very slender, threadlike	Bidentate hooked, with enlarged long spine on concave side of stem	Posterior chaetigers	South Africa
Aricidea (Aricidea) capensis bansei Laubier & Ramos, 1974	Elongate, longer than wide	When present, one pair (red)	Long, annulated, moniliform; to chaetiger 2	4 to 12–13	Chaetigers 1–2 rudimentary; well developed from chaetiger 3. Posterior: very long	Hooked, with 1-3 secondary teeth on principal tooth; with a subterminal spine on concave side of stem	22–27	Northwestern Mediterranean Sea; Adriatic Sea
Aricidea (Aricidea) curviseta Day, 1963	Blunted triangular	Absent	Short, not reaching the tip of prostomium	4 to 40	Prebranchial and branchial: short, conical; slender. Posterior: as slender filament	Thick stem directly transferring into slender spine	Postbranchial chaetigers	South Africa
Aricidea (Aricidea) fragilis Webster, 1879	Triangular, rounded anteriorly	One pair, small (usually not visible when preserved)	Short, subulate; to chaetiger 2	4 to 53–63	Chaetiger 1–2: short, digitiform. Chaetiger 3 and branchial: longer, wider basally. Posterior: digitiform to filiform	Pseudoarticulate, stouter basally, partially or completely fracturing at the midpoint	4–5 post-branchial chaetigers	Chesapeake Bay, off Eastern shore, Virginia
Aricidea (Aricidea) longicirrata Hartmann-Schröder, 1965	Pin shaped, tapered anteriorly	Absent	Short; to chaetiger 1	4 up to 17	Chaetiger 1–2: short, tuberculate. Chaetiger3: digitate. Branchial: threadlike. Posterior: shorter	Acicular, hooked, with slender subterminal spine on concave side of stem	13	Chile
Aricidea (Aricidea) longobranchiata Day, 1961	Roughly cordate, bluntly rounded anteriorly	Absent	Very long; to chaetiger 5	4 to 21	Prebranchial and branchial: cirriform with basal enlargement. Posterior: very short, slender	Acicular, hooked, with enlarged long spine on concave side of stem	Posterior chaetigers	South Africa
Aricidea (Aricidea) minima Strelzov, 1973	Elongated, conical, tapered anteriorly	Absent	Thickened; to chaetiger 2	4 to 19	Chaetigers 1–2: tuberculate. Chaetiger 3 and branchial: long with asymmetrical enlargement. Posterior: thin, longer.	Pseudoarticulate	Last branchial chaetiger	Patagonia, South America
Aricidea (Aricidea) minuta Southward, 1956	Conical	Absent	Short, bi- or triarticutlate; to chaetiger 1	4 to 16	Chaetiger 1–2: very short, tuberculate. Chaetiger3: digitiform, slender. Branchial small. Posterior: thinner	Pseudoarticulate	Unknown	Irish Sea and Baltic Sea
Aricidea (Aricidea) multiantennata Lovell, 2002	Triangular, bulbous end	Faded eyespot present	Five short tapering digitate branches	4 to 27–28	Prebranchial: digitate. Branchial and posterior: filiform	Pseudoarticulate, with a fringe on the convex side	37–39	Phuket, Andaman Sea, Thailand
Aricidea (Aricidea) petacalcoensis de León-González et al., 2006	Conical, rounded anteriorly	Absent	Short, bifurcate; to chaetiger 1	4 to 13–14	Chaetiger 1–2: absent. Chaetiger 3 and branchial: digitate. Posterior: increasing in size	Distally curved, with a subterminal spine on concave side of shaft	21	Western Mexico
Aricidea (Aricidea) pseudoarticulata Hobson, 1972	Triangular	Absent	Short, clavate with terminal papilla (bottle-shaped); chaetiger 1	4 to 14–16	Prebranchial: short. Branchial: longer, broad at base. Posterior: longer, cirriform	1) pseudoarticulate, long appendage; 2) tapered to hairlike tip; 3) hooked with hairlike tip; 4) hooked without hairlike tip	28–35	Southern California
Aricidea (Aricidea) rosea Reish, 1968	Triangular, rounded anteriorly	Absent	Slender; to chaetiger 2	4 to 14–15	Cirriform	Curved acicular with a subterminal spine on the concave side and pointed hood	Around 20–25	Los Angeles Bay, Gulf of California
Aricidea (Aricidea) sanmartini Aguado & López, 2003	Triangular, rounded anteriorly	Two pairs	Very long; to chaetiger 9	4 to 20	Chaetiger 1–2: very short. From chaetiger 3: strong, longer. Posterior: short, slender	Thick, hooked, with a very long subterminal spine	20–21	Coiba Island, Panama
Aricidea (Aricidea) thailandica Lovell, 2002	Triangular	One pair	With 2–3 pseudoarticulate branches, each with subdistal swelling tapering to filiform tip	4 to 18–24	Prebranchial: papillary, longer at chaetiger 3. Branchial: digitate. Posterior: filiform	Acicular, recurved tip, with terminal arista and hood emerging from concave side	34	Phuket, Andaman Sea, Thailand
Aricidea (Aricidea) wassi Pettibone, 1965	Conical, elongated	Absent	Long, articulate (12 articles); to chaetiger 4	4 to 13–21	Prebranchial: tuberculate. Branchial: cirriform. Posterior: very slender, threadlike	Acicular, hooked, with enlarged subterminal spine on concave side of stem	22–40	Northwestern Atlantic Ocean
Aricidea (Aricidea) thammapinanae sp. nov.	Conical, distally rounded	One pair	Short, biarticulate; to chaetiger 1	4 to 7–11	First two short, third larger, broadly triangular, with a short round distal protuberance; digitiform in branchial region; slender posteriorly	Bidentate hooked, with distal pubescence, with a very long subterminal spine on concave side of shaft	10–19	Songkhla Sea, Gulf of Thailand

The aim of this study is to examine in detail the morphological characteristics of these specimens using scanning electron microscope (SEM) images and light microscope photographs to verify differences from previously described species, and to confirm them as new species or not. Comparative tables of the diagnostic features of the new species and of those observed in closely similar taxa are included.

## Materials and methods

**Figure 1.**
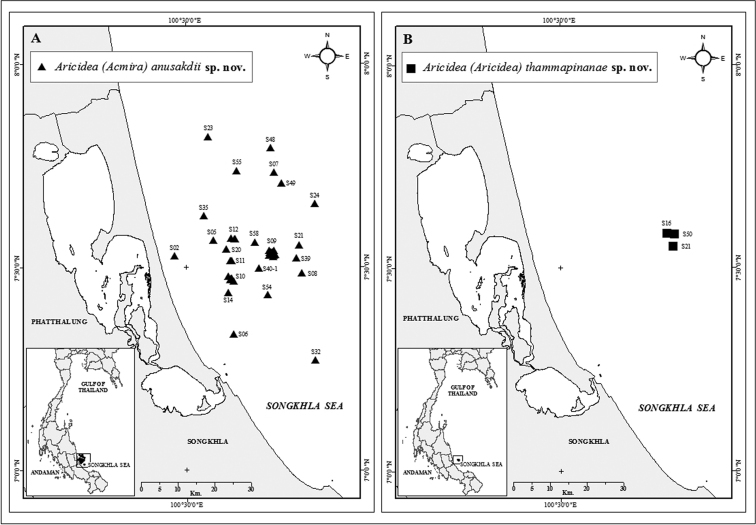
Sampling sites in the Songkhla Sea, Gulf of Thailand, showing stations where Aricidea (Acmira) anusakdii sp. nov. (**A**) and Aricidea (Aricidea) thammapinanae sp. nov. (**B**), were collected between 2011 and 2018.

Specimens were collected between 2011 and 2018 in the southern Gulf of Thailand (7°14'21"–7°49'21"N, 100°24'42"–100°49'00"E) (Fig. 1), with a Van Veen grab (0.1 m^2^) at depths ranging from 10 to 26.5 m. The collected samples were sieved with 2.0 mm, 1.0 mm and 0.5 mm mesh screens in the field. Later, water and sediment from the sieved grab samples were passed through a 300 µm filter bag. Specimens retained by both separation methods were separately fixed with a 4% formaldehyde in seawater solution. In the laboratory, samples were washed with fresh water and transferred to 70% ethanol. The polychaetes were sorted into taxonomic groups using a stereomicroscope and those belonging to the proposed new species were examined under dissection and compound light microscopes. SEM images were produced with a JEOL JSM-5800LV microscope and Apreo-Field Emission Scanning Electron Microscope (FESEM) at the Office of Scientific Instrument and Testing (OSIT), Prince of Songkla University, Hatyai, Thailand. Light microscope photographs were taken with a Leica digital camera in the OSIT, Prince of Songkla University and Olympus SZX16 with DP74 digital camera at MEM. The morphological measurements of the holotype are indicated in the taxonomic description. Information on character variability found in the paratypes is included in parentheses. The confirmation of the taxonomic status of the new species was based on the excellent revision and compilation of the diagnostic characteristics of all recognized species of the subgenera Aricidea (Acmira) and Aricidea (Aricidea) by Blake (1996), Lovell (2002) and Arriaga-Hernández et al. (2013). For comparative purposes, a table with the main diagnostic characters of the new species and closely related species was also included.

The type material was deposited in the Princess Maha Chakri Sirindhorn Natural History Museum, Prince of Songkla University (PSUZC), Thailand and the Australian Museum (AM), Sydney, Australia. Additional material is maintained in the personal collections of Jintana and Sakanan Plathong at MEM (Marine Ecosearch Management Co., Ltd.).

## Systematics

### Class Polychaeta

**Subclass Sedentaria**

**Infraclass Scolecida**

**Family Paraonidae Cerruti, 1909**

**Genus *Aricidea* Webster, 1879**

**Subgenus Aricidea (Acmira) Hartley, 1981**

#### Aricidea ( Acmira)  anusakdii sp. nov.

Taxon classificationAnimaliaCirratulidaParaonidae

6AA3B4E4-D8A3-5DBC-9257-BD0B2ECEFA9C

http://zoobank.org/5D4169E8-76CF-4638-8E77-70D681CCCF3B

**Figure 2.**
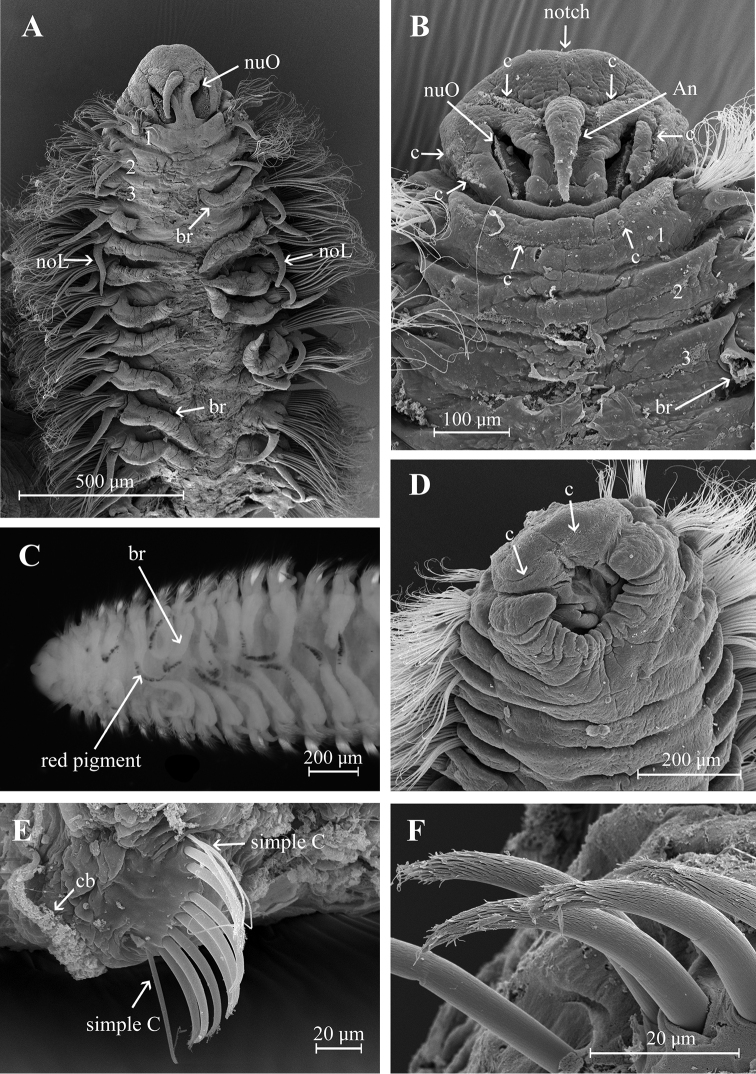
Aricidea (Acmira) anusakdii sp. nov. **A, B** anterior region, dorsal view **C** mouth,ventral view **D** branchial region, dorsal view **E** modified neurochaetae from posterior chaetiger **F** modified hooks. Abbreviations: An: antennae, br: branchia, c: cilia, cb: cilia band, noL: notopodial postchaetal lobe, nuO: nuchal organ, simple C: simple chaetae.

**Figure 3.**
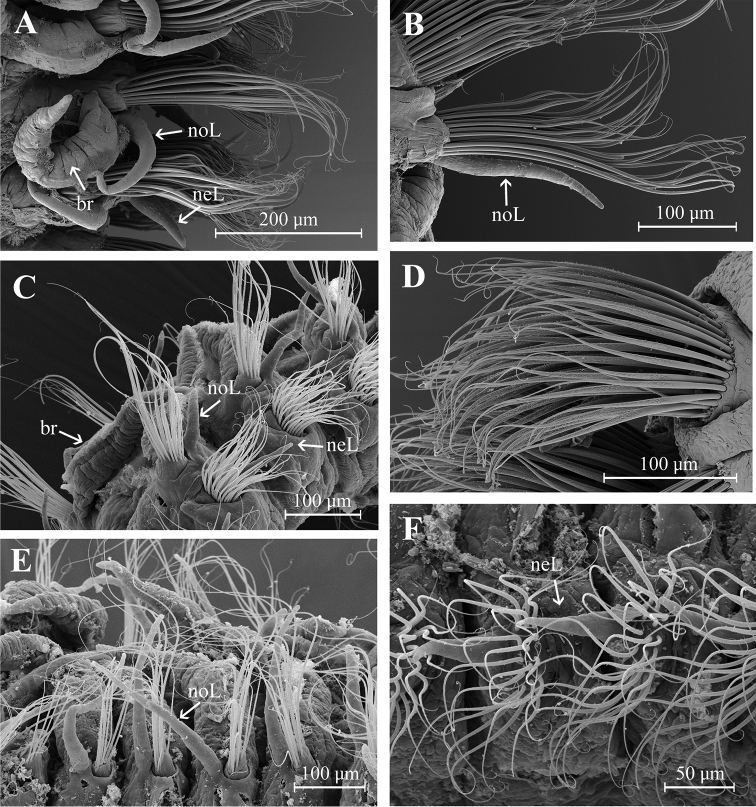
Aricidea (Acmira) anusakdii sp. nov. **A** branchiae at chaetiger 7 **B** notochaetae and notopodial postchaetal lobes (chaetiger 6) **C** noto- and neuropodial postchaetal lobes from chaetigers 13–14 **D** neurochaetae at chaetiger 14 **E** notopodial postchaetal lobes from midbranchial chaetiger **F** neuropodial postchaetal lobes and neurochaetae from midbranchial chaetiger. Abbbreviations: br: branchia, neL: neuropodial postchaetal lobe, noL: notopodial postchaetal lobe).

**Figure 4.**
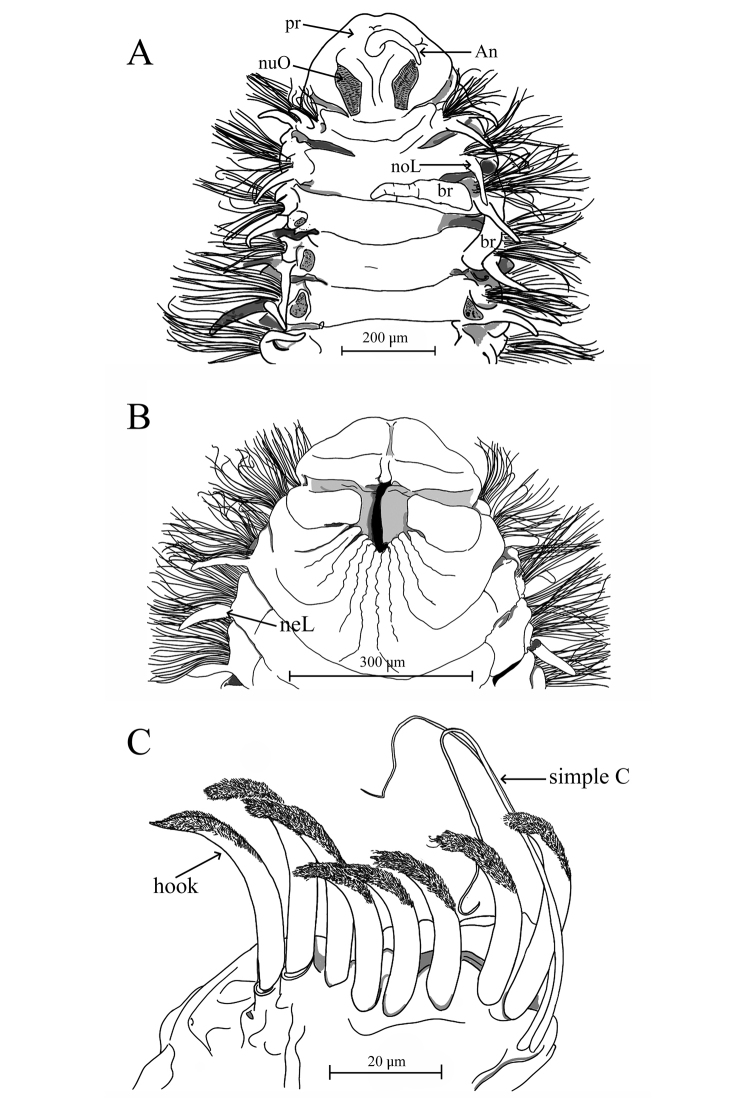
Aricidea (Acmira) anusakdii sp. nov. **A** anterior region, dorsal view **B** the buccal lip; ventral view **C** posterior modified neurochaetae. Abbreviations: An: antenna, br: branchia, neL: neuropodial postchaetal lobe, noL: notopodial postchaetal lobe, nuO: nuchal organ, pr: prostomium, simple C: simple chaetae).

Figures 1A , 2 , 3 , 4

##### Material examined.

178 specimens, incomplete, collected from Songkhla Sea, the Gulf of Thailand, Western Pacific. Coll. MEM (Marine Ecosearch Management Co., Ltd.), mud mixed with sand and shells. Details of geographic positions and environmental characteristics of sampling stations are in Table 2. ***Holotype*.**PSUZC-POL-0047, Sta. S09-24, 21 Mar. 2017. ***Paratypes*.**PSUZC-POL-0048 (1 spec.), Sta. S09-6, 4 Mar. 2011; PSUZC-POL-0049 (1 spec.), Sta. S07, 4 Jun. 2014; PSUZC-POL-0050 (1 spec.), Sta. S08, 4 Jun. 2014; PSUZC-POL-0051 (1 spec.), Sta. S10-3, 5 May 2018; PSUZC-POL-0052 (1 spec.), Sta. S07, 22 Sep. 2016; PSUZC-POL-0053 (1 spec., coated with gold for SEM), Sta. S05, 23 Mar. 2012; PSUZC-POL-0054 (2 specs., coated with gold for SEM), Sta. S07, 1 Jun. 2013; PSUZC-POL-0055 (1 spec.), Sta. S08, 14 Oct. 2015; PSUZC-POL-0056 (1 spec., coated with gold for SEM), Sta. S07, 16 Mar. 2016; PSUZC-POL-0058 (1 spec.), Sta. S07, 22 Sep. 2016; PSUZC-POL-0059 (1 spec.), Sta. S10-9, 6 May 2018; PSUZC-POL-0060 (1 spec.), Sta. S09-3, 16 Aug. 2018; AM W.52894 (1 spec.), Sta. S12-3, 9 Feb. 2012; AM W.52895 (1 spec.), Sta. S10-9, 6 May 2018.

**Table 2.** Stations, geographic positions, depths and sediment types where Aricidea (Acmira) anusakdii sp. nov. and Aricidea (Aricidea) thammapinanae sp. nov. were collected in the Songkhla Sea, Gulf of Thailand. (*= specimen used for SEM image and italic = A. (A.) thammapinanae).

Station	Sampling Date/ Number of individuals	Latitude / Longitude	Depth (m)	Sediment type
**S02**	11/10/2013 (1)	7°31'44"N, 100°28'15"E	10	Muddy with sand
**S05**	23/5/ 2012 (1*), 21/5/2015 (2)	7°34'03"N, 100°33'57"E	16.5	Muddy with sand and shells; upper sediment brown, lower sticky and dark
**S06**	17/10/2013 (1)	7°20'09"N, 100°36'58"E	15.5	Upper sediment muddy with sand, lower sticky mud with shells
**S07**	24/5/2012 (2), 10/10/2012 (1), 21/2/2013 (1), 1/6/2013 (5*), 16/10/2013 (3), 5/2/2014 (2), 4/6/2014 (3), 8/10/2014 (5), 26/2/2015 (1), 20/5/2015 (4), 20/5/2015 (2), 16/3/2016 (1*), 18/5/2016 (3), 22/9/2016 (5)	7°44'01"N, 100°43'02"E	26.5	Muddy with shells; upper sediment brown, lower sticky and green
**S08**	30/1/2012 (1), 24/5/2012 (2), 10/10/2012 (1), 1/6/2013 (1), 16/10/2013 (1), 5/2/2014 (4), 4/6/2014 (1), 8/10/2014 (1), 14/10/2015 (1*)	7°29'10"N, 100°47'06"E	25.0	Muddy with shells; lower sediment sticky mud
**S09-1**	7/2/2012 (1), 24/3/2017 (2), 17/8/2018 (2)	7°32'13"N, 100°42'41"E	24	Muddy with sand and shells
**S09-3**	7/3/2011 (1), 8/3/2014 (1), 16/8/2018 (2)	7°32'1"N, 100°42'41"E	24	Muddy with sand and shells
**S09-5**	17/8/2018 (2)	7°32'1"N, 100°42'30"E	24	Muddy with sand and shells
**S09-6**	4/3/2011 (1)	7°32'13"N, 100°42'21"E	23.6	Muddy with sand and shells
**S09-7**	7/2/2012 (1)	7°32'18"N, 100°42'24"E	23.7	Muddy with sand and shells
**S09-10**	7/3/2014 (1), 25/3/2017 (1)	7°31'55"N, 100°42'47"E	24.3	Muddy with sand and shells
**S09-11**	8/3/2014 (2), 25/3/2017 (1)	7°31'52"N, 100°42'42"E	23	Muddy with sand and shells
**S09-12**	6/2/2012 (1), 8/3/2014 (1), 25/3/2017 (2)	7°31'55"N, 100°42'24"E	23.8	Muddy with sand and shells
**S09-14**	1/3/2011 (2), 7/3/2014 (2), 1/3/2016 (2)	7°32'30"N, 100°42'12"E	24	Muddy with sand and shells
**S09-16**	1/3/2016 (2), 23/3/2017 (2)	7°32'30"N, 100°42'59"E	24	Muddy with sand and shells
**S09-17**	17/3/2013 (1), 7/3/2014 (2)	7°31'54"N, 100°43'5"E	24	Muddy with sand and shells
**S09-18**	6/2/2012 (1), 7/3/2014 (1)	7°31'44"N, 100°42'58"E	24	Muddy with sand and shells
**S09-19**	6/2/2012 (2), 7/3/2014 (1)	7°31'37"N, 100°42'48"E	24	Muddy with sand and shells
**S09-20**	6/2/2012 (2)	7°31'44"N, 100°42'12"E	24	Muddy with sand and shells
**S09-22**	16/8/2018 (1)	7°32'13"N, 100°42'30"E	24	Muddy with sand and shells
**S09-24**	7/2/2012 (1), 7/3/2014 (1), 21/3/2017 (1)	7°32'18"N, 100°42'47"E	24.5	Muddy with sand and shells
**S10**	2/3/2011 (1)	7°28'20"N, 100°36'33"E	19	Muddy with shells
**S10-3**	16/2/2015 (2), 5/5/2018 (2)	7°28'22"N, 100°36'41"E	19	Muddy with shells
**S10-4**	6/2/2012 (1), 15/2/2015 (3), 5/5/2018 (2)	7°28'14"N, 100°36'39"E	19	Muddy with shells
**S10-5**	6/2/2012 (4), 6/5/2018 (1)	7°28'12"N, 100°36'31"E	18.5	Muddy with shells
**S10-8**	5/2/2012 (3), 6/5/2018 (1)	7°28'43"N, 100°36'10"E	18.5	Muddy with shells
**S10-9**	16/2/2015 (2), 6/5/2018 (2)	7°27'57"N, 100°36'56"E	19	Muddy with shells
**S11-2**	27/3/2017 (1)	7°31'01"N, 100°36'39"E	18.9	Muddy with shells
**S11-3**	15/3/2013 (1), 27/3/2017 (1)	7°31'01"N, 100°36'27"E	18.8	Muddy with shells
**S12**	16/3/2013 (1)	7°34'18"N, 100°36'34"E	20	Muddy with sand and shells
**S12-2**	26/3/2017 (1)	7°34'12"N, 100°37'15"E	20	Muddy with sand and shells
**S12-3**	9/2/2012 (1)	7°34'13"N, 100°37'4"E	19.8	Muddy with sand and shells
**S14**	14/3/2013 (1), 5/3/2014 (1), 19/2/2015 (1)	7°26'13"N, 100°36'12"E	15.5	Muddy with sand and shells
**S16**	21/08/2012 (1*)	7°35'11"N, 100°45'47"E	22	Muddy with sand and shells
**S20**	20/8/2012 (2)	7°32'41"N, 100°35'54"E	21	Muddy with shells
**S21**	21/8/2012 (2), 23/3/2017 (4), 16/8/2018 (1), 21/08/2012 (1), 15/03/2013 (3, 1*), 3/06/2013 (1), 23/03/2017 (1), 23/09/2017 (1*), 16/08/2018 (4)	7°33'16"N, 100°46'43"E	24	Muddy with sand and shells
**S23**	29/2/2016 (1)	7°49'20"N, 100°33'17"E	20.5	Muddy with sand and shells
**S24**	30/10/2014 (1), 16/9/2014 (3), 30/10/2014 (1), 15/7/2015 (1)	7°39'22"N, 100°49'1"E	27	Fine mud with shells
**S32**	26/9/2011 (5)	7°16'18"N, 100°49'0"E	20	Muddy with shells
**S35**	29/9/2011 (1)	7°37'35"N, 100°32'35"E	24	Muddy with sand and shells
**S39**	27/9/2011 (2)	7°31'22"N, 100°46'15"E	22	Muddy sand
**S40-1**	20/8/2012 (1)	7°29'51"N, 100°40'41"E	20	Muddy sand
**S48**	22/2/2015 (1)	7°47'37"N, 100°42'29"E	24.6	Slightly muddy soil
**S49**	21/2/2015 (2)	7°42'25"N, 100°44'6"E	24.7	Slightly muddy soil
**S50**	27/02/2015 (1)	7°35'00"N, 100°46'57"E	24	Muddy with sand and shells, greenish brown
**S54**	21/2/2015 (2)	7°25'57"N, 100°42'2"E	15	Muddy with shells
**S55**	14/7/2015 (1)	7°44'16"N, 100°37'30"E	21	Muddy with shells
**S58**	30/9/2011 (2)	7°33'43"N, 100°40'10"E	20	Muddy with sand and shells

##### Description.

Holotype incomplete with 123 chaetigers, 25 mm long, 1.2 mm wide. Paratypes incomplete with 19–81 chaetigers, 3–13 mm long, 0.51–0.77 mm wide. Body robust, widest anteriorly, dorsoventrally flattened in branchial region (Fig. 2A), thinner with cylindrical segments in postbranchial region. Cilia scattered on dorsum along the body. Opaque white in alcohol, with red pigments on the distal and subdistal regions of each branchia (Fig. 2C). Prostomium wider than long (0.36 mm wide; 0.26 mm long); anterior margin of prostomium bilobed divided by a shallow notch which dorsally extends to the antenna (Figs 2B, 4A). Two large nuchal grooves on posterior half of prostomium; two ciliated bands on middle prostomium, and a ciliary band border on the inferior mid-region (Figs 2A, B, 4A). Short median antenna, proximally inflated, tapering to a short, blunt end, extending to posterior margin of prostomium (Figs 2A, B, 4A). No eyes. Anterior region of the mouth with a middle lobe and a ciliary row on its middle-anterior margin; posterior buccal lip with 12–14 small longitudinal folds, extending to chaetiger 2 (Figs 2D, 4B).

Two prebranchial chaetigers (Figs 2A, B, 4A). Branchiae start from chaetiger 3, 53 pairs (48–68 pairs in paratypes), bearing numerous long and slender cilia on dorsal midline (Fig. 3A); last pair of branchiae shorter. Parapodia large and thick with numerous simple chaetae on noto- and neuropodia. Notopodial postchaetal lobes from chaetiger 1 long, digitiform; cirriform in branchial and postbranchial chaetigers (Fig. 3B, E, F). Neuropodial postchaetal lobes shorter than notopodial postchaetal lobes (Fig. 3A, C, F).

Modified neurochaetae from chaetiger 37 (from 18–44 in paratypes) to posterior body region; up to nine modified chaetae per fascicle, each a curved spine with blunt shaft surrounded by pubescence (Fig. 2F), accompanied by about four simple chaetae on the superior and inferior parts of bundle (Figs 2E, 4C). All other chaetae long and slender capillaries (Figs 2C, 3D); notochaetae longer than neurochaetae (Fig. 3B, C). Pygidium unknown.

##### Reproduction.

Holotype and paratypes of A. (Acmira) anusakdii sp. nov. collected in March, May, June, and August had eggs in their branchial chaetigers. Eggs were also found in October in non-type material.

##### Etymology.

The species was named in honor of, and to remember, Mr Anusakdi Plathong, Sakanan’s deceased father.

##### Habitat.

At 10–26.5 m depth, in mud mixed with sand and shells substrates.

##### Distribution.

Songkhla Sea, Gulf of Thailand, Western Pacific.

##### Remarks.

Currently, the subgenus Aricidea (Acmira) Hartley, 1981 is represented by 20 species, including the new species described in the present study. The species that make up this subgenus can be separated by the features of modified neurochaetae (teeth, hood, distal arista, and pubescence), the length and shape of the median antenna, the number of prebranchial chaetigers and the number of branchiae (Arriaga-Hernández et al. 2013). Previously, only one species, A. (Acmira) simonae Laubier & Ramos, 1974, had been described with two prebranchial chaetigers. However, this taxon, originally described from Marseille, France, and common in Mediterranean and Black Sea is entirely different from the new species collected in Thailand. Aricidea (Acmira) simonae has smooth neuropodial spines, without pubescence, a very short antenna on the insertion area, bears only 20–32 pairs of branchiae and lacks neuropodial lobes. Aricidea (Acmira) anusakdii sp. nov. has curved spines with blunt shafts surrounded by pubescence, an antenna that reaches the posterior margin of the prostomium, has neuropodial lobes and bears a significantly higher number of branchial pairs (48–68 pairs).

Apart from A. (Acmira) simonae and A. (Acmira) anusakdii sp. nov., eight species of this genus also have smooth modified spines, lacking hood and distal arista, of which only A. (Acmira) hirsuta Arriaga-Hernández, Hernández-Alcántara & Solís-Weiss, 2013 from the southern Gulf of Mexico, A. (Acmira) horikoshi Imajima, 1973 from Japan and A. (Acmira) flava Zhou & Reuscher, 2013 from China, and probably A. (Acmira) simplex from South Africa and A. (Acmira) strelzovi from Antarctica, have modified spines with distal or subdistal pubescence. However, in these first three species the branchiae initially appear in chaetiger 4, bearing 7–15, 27 and 5 branchial pairs respectively. Clearly, these characteristics distinguish these species from A. (A.) anusakdii sp. nov., which, has two prebranchial chaetigers and a much greater number of branchiae (48–68 pairs). Aricidea (Acmira) anusakdii sp. nov. can also be separated from A. (Acmira) hirsuta because the new species has neuropodial lobes, which are absent in A. (Acmira) hirsuta (Table 3).

**Table 3.** Comparison of Aricidea (Acmira) species with modified spines lacking distal aristae and hood (modified from Arriaga-Hernández et al. 2013).

Character	Aricidea (Acmira) flava Zhou & Reuscher, 2013	Aricidea (Acmira) hirsutaArriaga-Hernández et al., 2013	Aricidea (Acmira) horikoshi Imajima, 1973	Aricidea (Acmira) simonae Laubier & Ramos, 1974	Aricidea (Acmira) anusakdii sp. nov.
Antenna (end at chaetiger)	3	Posterior margin of prostomium	4 to 5	Very short, on insertion area	Posterior margin of prostomium
Branchiae from chaetiger	4 to 21	4 to 10–18	4 to 33	3 to 20–32	3 to 48–68
Spines	Unidentate	Unidentate	Unidentate	Unidentate	Unidentate
Hood on spine	Absent	Absent	Absent (a narrow sheath on convex side)	Absent	Absent
Distal arista on spines	Absent	Absent	Absent	Absent	Absent
Pubescence on spines	Distal	Distal and subdistal	Distal	Absent	Distal and subdistal
Notopodial lobes	Present	Present	Present	Present	Present
Neuropodial lobes	Present (inconspicuous, low tubercles)	Absent	Present	Absent	Present
Type locality	Northern coast of China	Términos Lagoon, southern Gulf of Mexico	Japan, North Pacific Ocean	Famagusta Bay, Marseille, France	Songkhla Sea, Gulf of Thailand

Although the modified spines in A. (Acmira) mirifica and A. (Acmira) finitima have no hood and do not bear distal or subdistal pubescence, in the first species the spines sometimes bear a short distal arista and in the second they almost always bear arista. Nonetheless, both these species can also be separated from the new species because they have three prebranchial chaetigers, their antennae are longer (reaching chaetiger 1–3 or 6), and they bear fewer branchiae, 12 and 14–27 pairs, respectively.

It is important to note that previously, the presence of lobes and notches on the anterior margin of the prostomium had only been reported in two species: A. (Acmira) simonae, which has three lobes in ventral view (Laubier and Ramos 1973) and other differences, smooth neuropodial spines, a very short antenna on the insertion area, bears only 20–32 pairs of branchiae and lacks neuropodial lobes, with the new species has been previously argued; and Aricidea (Acmira) trilobata Imajima, 1973, distributed on the continental shelves of Japan and California (Blake, 1996), which also bears three lobes on the anterior edge of the prostomium and the branchiae start from chaetiger 4. However, unlike the new species, this last species also bears three lobes on the anterior edge of the prostomium, the branchiae start from chaetiger 4, the median antenna extending to chaetiger 2 and only bears 18 to 20 branchial pairs.

### Genus *Aricidea* (Webster, 1879)

**Subgenus Aricidea (Aricidea) [Webster, 1879, sensu stricto]**

#### Aricidea ( Aricidea)  thammapinanae sp. nov.

Taxon classificationAnimaliaCirratulidaParaonidae

53DA22AA-FE2C-581A-9741-5ED999DDADAC

http://zoobank.org/6B8798D3-662C-4097-8DC9-83A5640E332C

**Figure 5.**
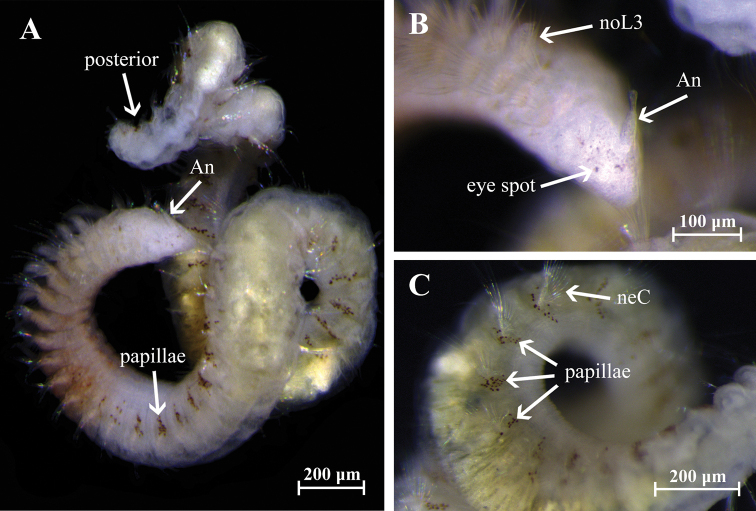
Aricidea (Aricidea) thammapinanae sp. nov. PSUZC 0047, holotype. **A** complete specimen, ventral view **B** prostomium, leteral view **C** red-brown papillae and neurochaetae, lateral view. Abbreviations: An: median antenna, neC: neurochaetae, noL3: notopodial postchaetal lobe chaetiger 3.

**Figure 6.**
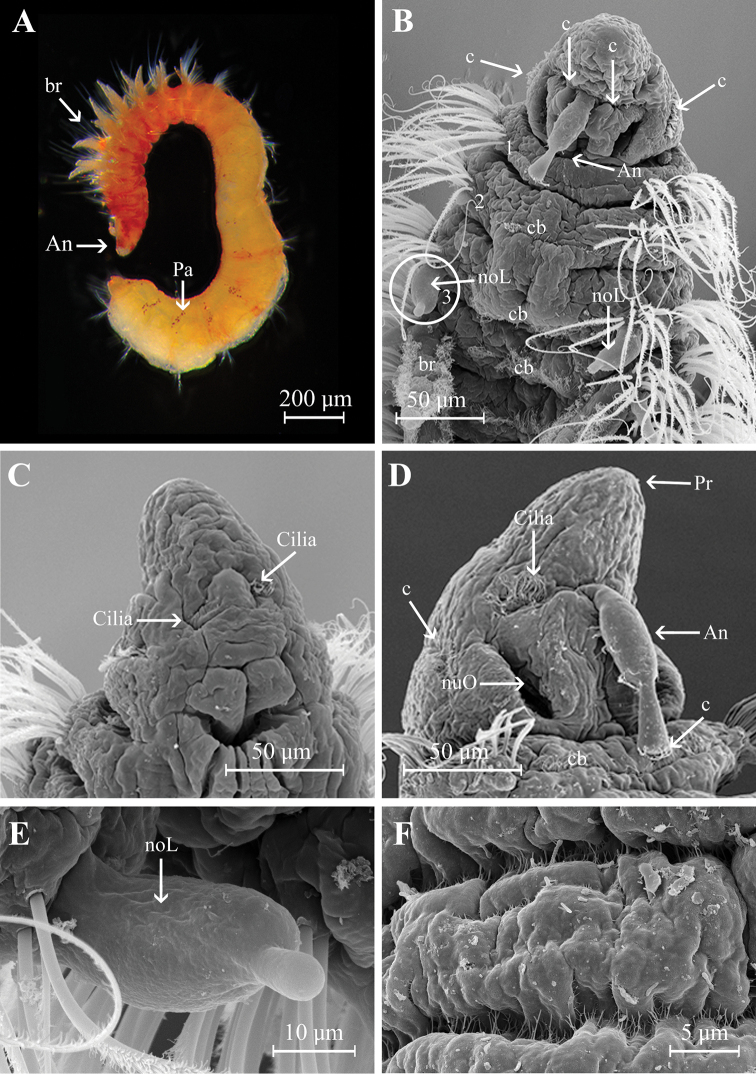
Aricidea (Aricidea) thammapinanae sp. nov. **A** body color in alcohol, lateral view **B** anterior region, dorsal view **C** mouth, arrows show cilia **D** prostomium, dorso-lateral view **E** notopodial lobe from chaetiger 3 **F** close up of posterior chaetiger, showing the cilia. Abbreviations: An: antenna, br: branchiae, c: cilia, cb: cilia band, noL: notopodial postchaetal lobe, nuO: nuchal organ, Pr: prostomium.

**Figure 7.**
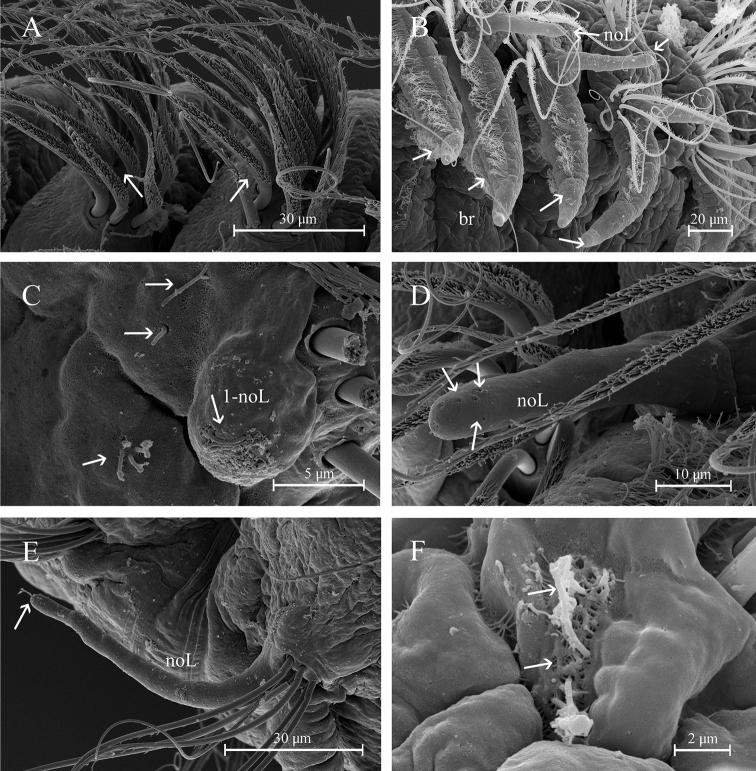
Aricidea (Aricidea) thammapinanae sp. nov. **A** three rows of neurochaetae in prebranchial area, ventral view **B** branchiae with long cilia **C** first notopodial lobe **D, E** pores on the distal area of notopodial lobe **E** notopodial lobe from chaetiger 3 **F** filaments or cilia in notopodial pores.

**Figure 8.**
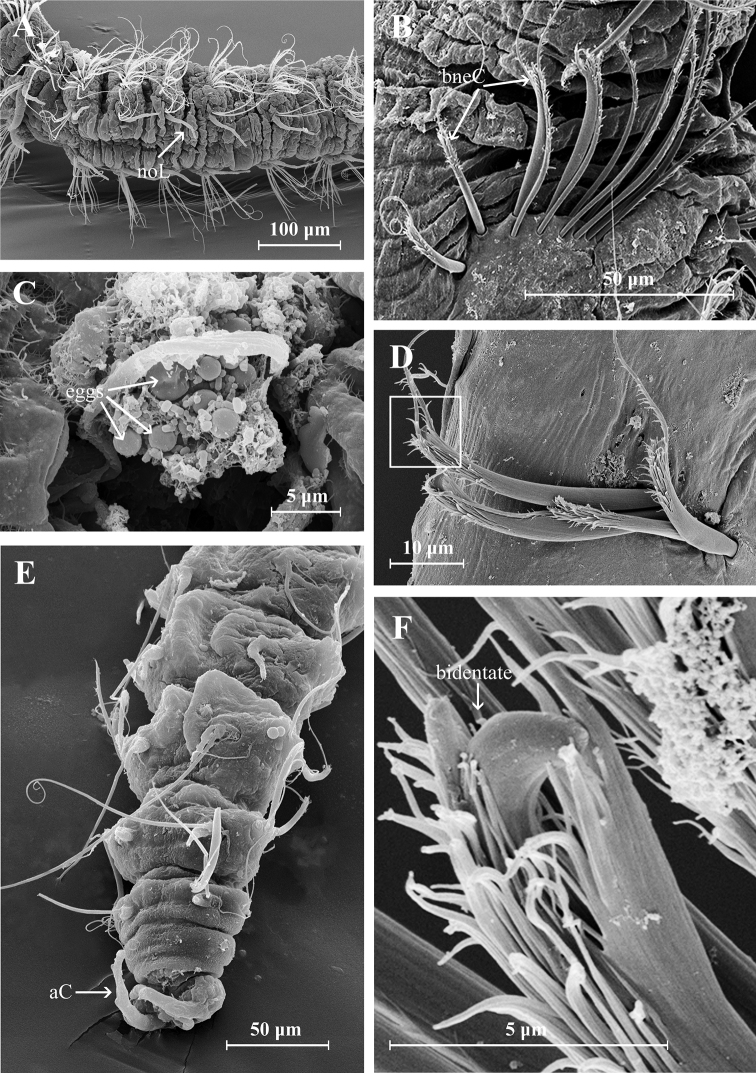
Aricidea (Aricidea) thammapinanae sp. nov. **A** postbranchial region, arrow shows the notopodial postchaetal lobe **B, D, F** modified neurochaetae **C** eggs in postbranchial region **E** posterior region, pygidium with two anal cirri. Abbreviations: aC: anal cirri, bneC: bidentate neurochaetae, noL: notopodial postchaetal lobe.

**Figure 9.**
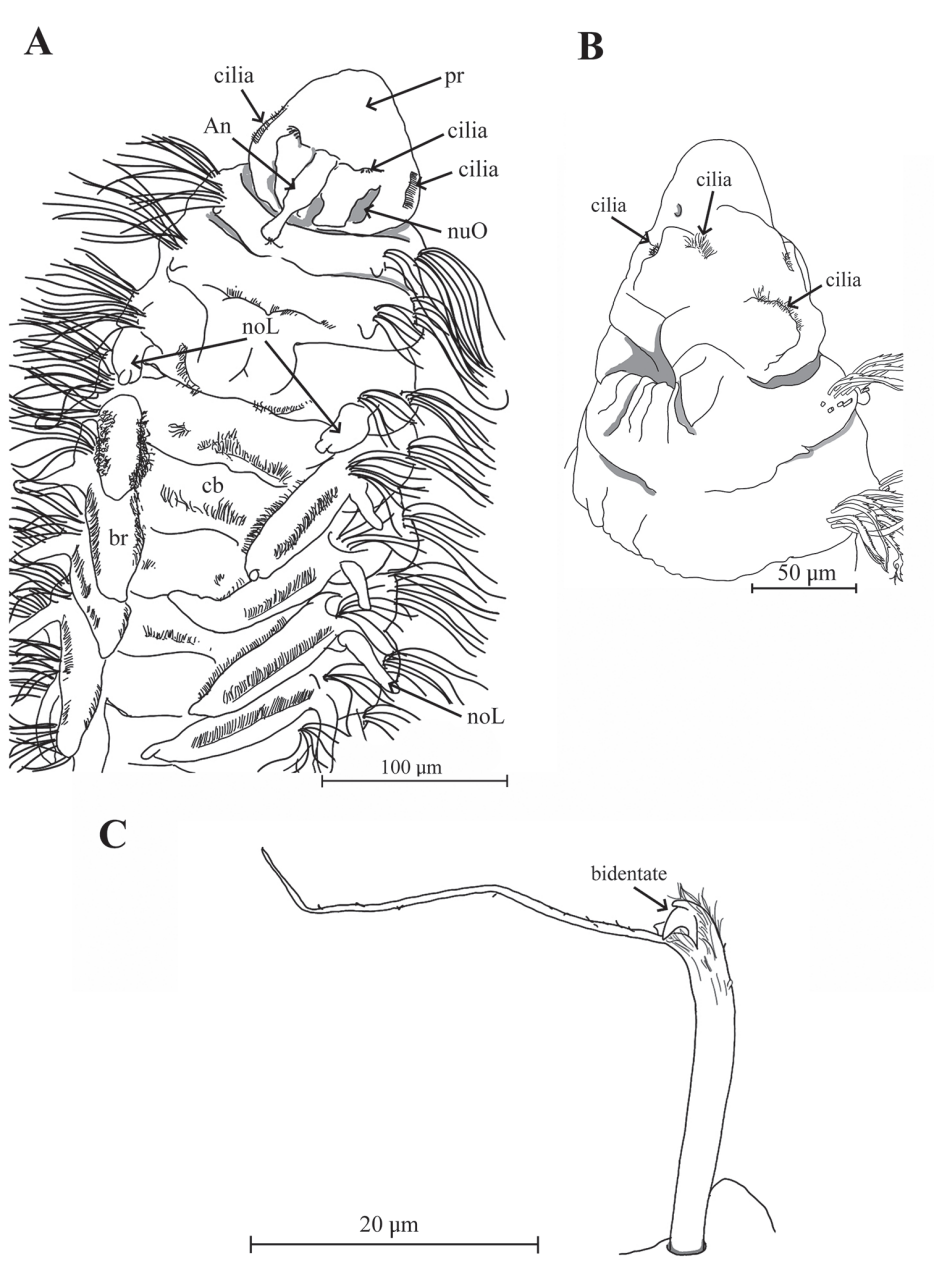
Aricidea (Aricidea) thammapinanae sp. nov. **A** anterior region, dorsal view **B** the buccal lip; lateral view **C** posterior modified neurochaetae, lateral view. Abbreviations: An: antenna, br: branchiae, cb: cilia band, noL: notopodial postchaetal lobe, nuO: nuchal organ, pr: prostomium.

Figures 1B , 5 , 6 , 7 , 8 , 9

##### Material examined.

13 specimens, collected from Songkhla Sea, Gulf of Thailand, 24 m depth. Coll. MEM (Marine Ecosearch Management Co., Ltd.), in mud mixed with sand and shells. Details of geographic positions and environmental characteristics of sampling stations are in Table 2. ***Holotype.***PSUZC-POL-00021 (1 spec., complete), Sta. S21, 16 Aug. 2018. ***Paratypes.***PSUZC-POL-00022 (1 spec.), Sta. S21, 21 Aug. 2012; PSUZC-POL-00023, (1 spec., coated with gold for SEM), Sta. S21, 15 Mar. 2013; PSUZC-POL-00024 (1 spec.), Sta. S21, 3 Jun. 2013; PSUZC-POL-00025 (1 spec.), 23 Mar. 2017; PSUZC-POL-00026, (1 spec., coated with gold for SEM), Sta. S21, 23 Sep. 2017; PSUZC-POL-0027, (1 spec., coated with gold for SEM), Sta. S16, 21 Aug. 2012; PSUZC-POL-0062 (2 specs.), Sta. S21, 16 Aug. 2018; AM W.52904 (1 spec.), Sta. S50, 27 Feb. 2015.

##### Description.

Holotype complete with approximately 50 chaetigers (posterior region coiled, difficult to count segments), 5.47 mm long, 0.3 mm wide (Fig. 5A–C); two complete paratypes with 29 and 45 chaetigers, others incomplete with 21 to 32 chaetigers, 1.8–4.5 mm long and 0.01–0.23 mm wide. Body small, new preserved specimens reddish-orange in prebranchial and branchial regions (Fig. 5A); dorsal ciliary bands present on the prebranchial and branchial chaetigers. Prostomium conical, distally rounded, longer than wide; one pair of small black or brown eyes present; two pairs of long ciliary bands, one pair located above nuchal grooves and other at lateral margins of prostomium. Median antenna biarticulated, basal portion clavate, distal portion triangular, ciliated on distal end; basal portion of median antenna about two times longer than distal portion, extending to chaetiger 1 (Figs 6B, D, 9A). Nuchal organs as pair of oblique, deep slits. Posterior buccal lip with six longitudinal folds, extending to chaetiger 1, with one pair of ciliary patches above the buccal region (Figs 6C, 9B). Numerous small filaments along body, and thin papillae present on the body (Fig. 6F) and notopodial pores (Fig. 7F).

Postbranchial region presents numerous dark red or brown pigmented papillae adjacent to neurochaetal rami on all chaetigers (Fig. 5A, C). First two notopodial postchaetal lobes very short, usually hidden by chaetae; those of chaetiger 3 much larger, broadly triangular, with a short, rounded distal protuberance. Notopodial postchaetal lobes digitate on branchial segments, filiform on following segments. Neuropodial postchaetal lobes small, inconspicuous (Figs 6B, E, 9A).

Three prebranchial chaetigers; 8 pairs of branchiae (4 to 8 in paratypes) present on chaetigers 4 to 11, robust, conical, with lateral margins markedly ciliated; last pair smaller. Anterior noto- and neurochaetae fringed with capillaries (Fig. 6A, B); notochaetae longer than neurochaetae, decreasing in number from anterior to posterior segments. Modified neurochaetae bidentate (Fig. 8B, E, F), beginning in chaetiger 17 (10–19 in paratypes), superior tooth small, inferior tooth large, surrounded by pubescence on distal region of shaft, with very long subterminal spine arising from concave side of shaft; spine almost twice as long as shaft, with pubescence throughout, starting from chaetiger 10–19. Posterior neurochaetae arranged in two rows, first row with both simple and modified bidentate chaetae, and second row with only simple chaetae; up to 5 bidentate chaetae per fascicle per row, accompanied by 10–12 long capillary chaetae (Fig. 8B). Pygidium with three anal cirri, two lateral and one triangular, short mid-ventral (Fig. 8E).

##### Reproduction.

Holotype and paratypes of Aricidea (Aricidea) thammapinanae sp. nov. collected in March, August, and September had eggs in the coelomic cavities of postbranchial chaetigers.

##### Etymology.

The species epithet *thammapinanae*, is after the family name of Ms Vorramaz Thammapinan. This species is named in honor of her initiation, coordination, and assistance to the research project in Songkhla Sea.

##### Habitat.

At 20–24 m depth, mud with sand and shells.

##### Distribution.

Songkhla Sea, Gulf of Thailand, Western Pacific.

##### Remarks.

This is a small species of the subgenus Aricidea (Aricidea) having a maximum length of 5.47 mm (holotype) and with only 4–8 pairs of branchiae. The presence of eggs (Fig. 7C) in individuals collected during several sampling months implies that the small size of this new taxon is a specific characteristic. The presence of bidentate chaetae is unusual in species belonging to the subgenus Aricidea (Aricidea). Until now, 15 species have been described in this subgenus but only Aricidea (Aricidea) capensis Day, 1961 from South Africa (Day 1961) has bidentate modified chaetae (Table 1). However, the species presents clearly different characteristics from those observed in A. (Aricidea) thammapinanae sp. nov., since the bidentate modified chaetae of A. (Aricidea) capensis Day, 1961 are smooth, without pubescence along the shaft or on the subterminal spine. Besides the antenna, extending to chaetiger 2, is faintly annulated, eyes are lacking, 14 branchial pairs are present, and all prebranchial notopodial lobes are small and slender (Table 1). In contrast, the proposed new species has bidentate modified neurochaetae with pubescence on the distal shaft and along the subterminal spine, a biarticulated antenna that extends to chaetiger 1, and a pair of eyes. Only 4–8 branchial pairs are present, and on chaetiger 3, distinctive broad triangular notopodial lobes with short distal protuberances.

## Supplementary Material

XML Treatment for Aricidea ( Acmira)  anusakdii

XML Treatment for Aricidea ( Aricidea)  thammapinanae
